# Association between characteristics of employing healthcare facilities and healthcare worker infection rates and psychosocial experiences during the COVID-19 pandemic

**DOI:** 10.1186/s12913-024-11109-6

**Published:** 2024-05-24

**Authors:** Jay B. Lusk, Pratik Manandhar, Laine E. Thomas, Emily C. O’Brien

**Affiliations:** 1 Department of Neurology, Duke University, DUMC 3710, Durham, NC USA; 2 Department of Population Health Sciences, Duke University, Durham, NC USA; 3 Duke University Clinical Research Institute, Durham, NC USA

**Keywords:** Occupational health, COVID-19, Hospital ownership, Non-profit, Burnout, Hospital size, Healthcare workers, Pandemic resilience

## Abstract

**Background:**

Healthcare facility characteristics, such as ownership, size, and location, have been associated with patient outcomes. However, it is not known whether the outcomes of healthcare workers are associated with the characteristics of their employing healthcare facilities, particularly during the COVID-19 pandemic.

**Methods:**

This was an analysis of a nationwide registry of healthcare workers (the Healthcare Worker Exposure Response and Outcomes (HERO) registry). Participants were surveyed on their personal, employment, and medical characteristics, as well as our primary study outcomes of COVID-19 infection, access to personal protective equipment, and burnout. Participants from healthcare sites with at least ten respondents were included, and these sites were linked to American Hospital Association data to extract information about sites, including number of beds, teaching status, urban/rural location, and for-profit status. Generalized estimating equations were used to estimate linear regression models for the unadjusted and adjusted associations between healthcare facility characteristics and outcomes.

**Results:**

A total of 8,941 healthcare workers from 97 clinical sites were included in the study. After adjustment for participant demographics, healthcare role, and medical comorbidities, facility for-profit status was associated with greater odds of COVID-19 diagnosis (aOR 1.76, 95% CI 1.02–3.03, *p* = .042). Micropolitan location was associated with decreased odds of COVID-19 infection after adjustment (aOR = 0.42, 95% CI 0.24, 0.71, *p* = .002. For-profit facility status was associated with decreased odds of burnout after adjustment (aOR = 0.53, 95% CI 0.29–0.98), *p* = .044).

**Conclusions:**

For-profit status of employing healthcare facilities was associated with greater odds of COVID-19 diagnosis but decreased odds of burnout after adjustment for demographics, healthcare role, and medical comorbidities. Future research to understand the relationship between facility ownership status and healthcare outcomes is needed to promote wellbeing in the healthcare workforce.

**Trial registration:**

The registry was prospectively registered: ClinicalTrials.gov Identifier (trial registration number) NCT04342806, submitted April 8, 2020.

**Supplementary Information:**

The online version contains supplementary material available at 10.1186/s12913-024-11109-6.

## Background

The COVID-19 pandemic added a major stressor to the already overtaxed healthcare system in the United States. Existing trends toward health system consolidation and from independent practice were exacerbated by the financial impact of the pandemic [1–3]. Prior work has demonstrated a consistent impact of healthcare facility characteristics on care quality, with variation in patient outcomes according to for-profit status, bed size, and teaching status; consolidation of health systems has also been linked with worsened patient outcomes [4–6].

There is also a robust literature relating health facility characteristics with the experiences of healthcare workers who work in those facilities. Several dimensions of the healthcare workplace are associated with outcomes for healthcare professionals. For example, there is an extensive literature demonstrating that physical environment (comprising items ranging from noise levels to temperature to ergonomics) is associated with workplace satisfaction and healthcare worker wellbeing [7]. Work environments that promote work engagement and self-efficacy are associated with job satisfaction and decrease turnover; by contrast, poor management, unprofessional behavior from colleagues, and unfavorable clinical work structures are associated with decreased job satisfaction and increased turnover [8–10]. Other workplace characteristics, such as high patient volumes, working a high burden of night shift work, and having a principally academic practice have been associated with increased burnout [11].

The operating characteristics of healthcare facilities may have a major influence on their ability to provide positive workplace environments for healthcare workers. For example, a prior study showed that registered nurses working in small hospitals were more likely to report job satisfaction than nurses working in large hospitals [12]. This same study found no association between employment in an academic hospital or facility location in a high population density area and satisfaction with the work environment, and no association with any of the above features and burnout after adjustment for age, sex, and level of education [12]. On the converse, a study of physical and occupational therapists found that employment in smaller healthcare facilities was associated with higher job stress and burnout levels [13]. A systematic review and meta-analysis of burnout by inpatient versus outpatient work environment of physicians found that outpatient physicians reported more emotional exhaustion than inpatient physicians, but otherwise did not find compelling evidence of differences in other psychosocial outcomes [14].

Despite the robust literature exploring the impact of employing healthcare facilities on outcomes of healthcare workers, this has not been explored in a large, diverse cohort of healthcare workers in the context of the COVID-19 pandemic. Therefore, our objective was to characterize the associations between healthcare facility characteristics and healthcare worker outcomes, including burnout, depression, and COVID-19 exposure and diagnosis, among participants of the Healthcare Worker Exposures, Response, and Outcomes (HERO) registry.

## Methods

The registry and this analysis (ClinicalTrials.gov Identifier NCT04342806) was funded by the Patient-Centered Outcomes Research Institute (PCORI) and was approved by the WIRB-Copernicus Group Institutional Review Board (WCG IRB). Characteristics of the registry and assessment tools have been described previously [15, 16]. Written informed consent was obtained from all participants. This study adhered to the tenets of the Declaration of Helsinki. We presented results according to the Strengthening the Reporting of Observational Studies in Epidemiology (STROBE) Statement. We analyzed data from participants enrolled in the HERO registry from April 2020-May 2022. Participants who did not provide information on their employing healthcare facility, or whose employing healthcare facility could not be linked to American Hospital Association database were excluded. Participants from any site with more than 10 healthcare workers (HCWs) enrolled were included, for a final cohort of 8,941 HCWs from 97 clinical sites. Exposures of interest included healthcare facility characteristics, namely bed size, teaching status, urban location, and for-profit status, obtained from American Hospital Association data. We evaluated associations between these characteristics and four outcomes of interest prioritized by HCW participants during the COVID-19 pandemic. These included 1) access to personal protective equipment (PPE) quantified on a summary scale from ten questionnaires asking about various surrogates of access to PPE, 2) COVID-19 diagnosis on at least one survey, 3) burnout (defined as responding to at least three burnout symptoms on a burnout instrument on at least one occasion), and 4) depression (defined as a PROMIS-T score > 60 on at least one survey). Generalized Estimating Equations—to account for within-site clustering— were used to estimate linear regression models for the access to PPE outcome, and to estimate logistic regression models for the other outcomes. Adjusted regression models included covariates for age, gender, race, ethnicity, role in healthcare setting (e.g. nurse), healthcare environment (e.g. outpatient), and self-reported medical comorbidities. Dependent and independent variables used in our study are described in detail in Appendix 1. Median values were used to impute missing continuous adjustment variables and mode values were used to impute missing categorical adjustment variables. Around 10% of participants were missing information on self-reported medical history, and < 1% of all other variables were missing. The vast majority of missing data on self-reported medical history was missing completely at random due to a version change in the data collection form. Imputation was only performed on missing adjustment variables and not on study endpoints. Unadjusted and adjusted odds ratios with 95% confidence intervals and p-values were estimated.

## Results

### Characteristics of the study population

Characteristics of included participants are shown in Table 1. The median age was 40 (IQR 33–51), 77.2% of the participants were female, 86.8% identified as White, 4.3% identified as Black or African American, and 8.9% identified as another race; 6.2% of participants identified as Hispanic ethnicity. Most participants worked in inpatient settings (80.2%); 9.2% worked in outpatient settings, 1.2% in emergency services, skilled nursing, or urgent care, and 9.4% in other healthcare settings. Nurses made up a plurality of the participants (32.3%) followed by physicians (21.9%), administrative staff (7.3%), and physicians assistants or nurse practitioners (6.2%).

**Table 1:** Characteristics of included participants

**Characteristic**	Category	N (%) or Median (IQR)
Age (Median, IQR)		40.0 (33.0, 51.0)
Gender, n (%)	Male	2035 (22.8)
	Female	6906 (77.2)
Race, n (%)	Black/African American	383 (4.3)
	Other race	795 (8.9)
	White	7763 (86.8)
Hispanic or Latino Ethnicity, n (%)	Prefer not to answer	92 (1.0)
	Yes	550 (6.2)
	No	8299 (92.8)
Healthcare Environment, n (%)	Other	838 (9.4)
	Outpatient	826 (9.2)
	Skilled Nursing Facility, Urgent Care, or Emergency Medical Services	107 (1.2)
	Hospital	7170 (80.2)
Role in Healthcare Setting, n (%)	Other	1418 (15.9)
	Medical Assistant	411 (4.6)
	Paramedic/Emergency Medical Technician	112 (1.3)
	Dietary/Nutrition/Food Services/Environmental Services	155 (1.7)
	Respiratory Therapist	116 (1.3)
	Administrative staff	650 (7.3)
	Physical therapist	139 (1.6)
	Lab technician, Pharmacist, Pharmacy technician	454 (5.1)
	Physician Assistant/Nurse Practitioner	557 (6.2)
	Physician, Physician-in-training	1961 (21.9)
	Nurse (RN/LPN)	2890 (32.3)
	Missing	78 (0.9)

### Characteristics of the study population stratified by characteristics of employing healthcare facilities

Baseline characteristics of the participants varied according to hospital characteristics. Table 2 shows baseline characteristics stratified by hospital ownership status. In summary, for-profit and not-for-profit sites had a somewhat higher proportion of workers who identified as non-Hispanic compared to government sites; furthermore, a greater share of respondents from not-for-profit hospitals were physicians or physicians in training.

**Table 2:** Baseline characteristics of participants stratified by ownership status of the employing healthcare facility

	Level	Overall *N* = 8,941 from 97 sites	Not-for-Profit *N *= 7,262 from 76 sites	For-Profit *N* = 104 from 5 sites	Government *N* = 1,575 from 16 sites	***P-***value
Age (Median, IQR)		40.0 (33.0-51.0)	41.0 (33.0-51.0)	42.0 (35.0-49.5)	40.0 (33.0-50.0)	0.086
Gender, n (%)	Male	2035 (22.8)	1683 (23.2)	18 (17.3)	334 (21.2)	0.099
	Female	6906 (77.2)	5579 (76.8)	86 (82.7)	1241 (78.8)	
Race, n (%)	Black/African American	383 (4.3)	333 (4.6)	2 (1.9)	48 (3.0)	0.018
	Other race	795 (8.9)	662 (9.1)	8 (7.7)	125 (7.9)	
	White	7763 (86.8)	6267 (86.3)	94 (90.4)	1402 (89.0)	
Hispanic or Latino Ethnicity, n (%)	Prefer not to answer	92 (1.0)	76 (1.0)	0 (0.0)	16 (1.0)	0.005
	Yes	550 (6.2)	458 (6.3)	14 (13.5)	78 (5.0)	
	No	8299 (92.8)	6728 (92.6)	90 (86.5)	1481 (94.0)	
Healthcare Environment, n (%)	Other	838 (9.4)	692 (9.5)	4 (3.8)	142 (9.0)	0.105
	Outpatient	826 (9.2)	653 (9.0)	6 (5.8)	167 (10.6)	
	Skilled Nursing Facility, Urgent Care, or Emergency Medical Services	107 (1.2)	85 (1.2)	1 (1.0)	21 (1.3)	
	Inpatient	7170 (80.2)	5832 (80.3)	93 (89.4)	1245 (79.0)	
Role in Healthcare Setting, n (%)	Other	1418 (15.9)	1130 (15.6)	15 (14.4)	273 (17.3)	< .001
	Medical Assistant	411 (4.6)	331 (4.6)	4 (3.8)	76 (4.8)	
	Paramedic/Emergency Medical Technician	112 (1.3)	94 (1.3)	1 (1.0)	17 (1.1)	
	Dietary/Nutrition/Food Services/Environmental Services	155 (1.7)	126 (1.7)	4 (3.8)	25 (1.6)	
	Respiratory Therapist	116 (1.3)	91 (1.3)	0 (0.0)	25 (1.6)	
	Administrative staff	650 (7.3)	535 (7.4)	5 (4.8)	110 (7.0)	
	Physical therapist	139 (1.6)	119 (1.6)	1 (1.0)	19 (1.2)	
	Lab technician, Pharmacist, Pharmacy technician	454 (5.1)	382 (5.3)	3 (2.9)	69 (4.4)	
	Physician Assistant/Nurse Practitioner	557 (6.2)	448 (6.2)	5 (4.8)	104 (6.6)	
	Physician, Physician-in-training	1961 (21.9)	1526 (21.0)	14 (13.5)	421 (26.7)	
	Nurse (RN/LPN)	2890 (32.3)	2416 (33.3)	49 (47.1)	425 (27.0)	
	Missing	78 (0.9)	64 (0.9)	3 (2.9)	11 (0.7)	

Table 3 shows baseline characteristics of the study population stratified by metropolitan versus micropolitan location; participants employed by facilities in micropolitan locations tended to be older, were more likely to identify as White and less likely to identify as Hispanic, were more likely to report employment in facilities other than hospitals and were more likely to be employed as medical assistants or medical administrators rather than physicians or registered nurses.

**Table 3:** Baseline characteristics of participants stratified by micropolitan versus metropolitan location of employing healthcare facilities

	Level	Overall *N* = 8,941 from 97 sites	Metropolitan *N* = 8,561 from 93 sites	Micropolitan *N* = 380 from 4 sites	***P-***value
Age (Median, IQR)		40.0 (33.0, 51.0)	40.0 (33.0, 51.0)	43.0 (35.0, 54.0)	0.002
Gender, n (%)	Male	2035 (22.8)	1955 (22.8)	80 (21.1)	0.417
	Female or other	6906 (77.2)	6606 (77.2)	300 (78.9)	
Race, n (%)	Black/African American	383 (4.3)	382 (4.5)	1 (0.3)	< .001
	Other race	795 (8.9)	775 (9.1)	20 (5.3)	
	White	7763 (86.8)	7404 (86.5)	359 (94.5)	
Hispanic or Latino Ethnicity, n (%)	Prefer not to answer	92 (1.0)	87 (1.0)	5 (1.3)	< .001
	Yes	550 (6.2)	546 (6.4)	4 (1.1)	
	No	8299 (92.8)	7928 (92.6)	371 (97.6)	
Healthcare Environment, n (%)	Other	838 (9.4)	773 (9.0)	65 (17.1)	< .001
	Outpatient	826 (9.2)	722 (8.4)	104 (27.4)	
	Skilled Nursing Facility, Urgent Care, or Emergency Medical Services	107 (1.2)	96 (1.1)	11 (2.9)	
	Inpatient	7170 (80.2)	6970 (81.4)	200 (52.6)	
Role in Healthcare Setting, n (%)	Other	1418 (15.9)	1348 (15.7)	70 (18.4)	< .001
	Medical Assistant	411 (4.6)	375 (4.4)	36 (9.5)	
	Paramedic/Emergency Medical Technician	112 (1.3)	107 (1.2)	5 (1.3)	
	Dietary/Nutrition/Food Services, Environmental Services	155 (1.7)	145 (1.7)	10 (2.6)	
	Respiratory Therapist	116 (1.3)	111 (1.3)	5 (1.3)	
	Administrative staff	650 (7.3)	614 (7.2)	36 (9.5)	
	Physical therapist	139 (1.6)	133 (1.6)	6 (1.6)	
	Lab technician, Pharmacist, Pharmacy technician	454 (5.1)	421 (4.9)	33 (8.7)	
	Physician Assistant/Nurse Practitioner	557 (6.2)	535 (6.2)	22 (5.8)	
	Physician, Physician-in-training	1961 (21.9)	1896 (22.1)	65 (17.1)	
	Nurse (RN/LPN)	2890 (32.3)	2801 (32.7)	89 (23.4)	
	Missing	78 (0.9)	75 (0.9)	3 (0.8)	

Table 4 shows baseline characteristics of the study population stratified by teaching status of the employing healthcare facility. Overall, participants from teaching institutions were younger, were less likely to identify as White and more likely to identify as Hispanic, were more likely to work in inpatient settings, and were more likely to be employed as physicians or nurses.

**Table 4:** Baseline characteristics of participants stratified by teaching status of employing healthcare facility

	Level	Overall *N *= 8,941 from 97 sites	Teaching = Yes *N *= 7,883 from 75 sites	Teaching = No *N* = 1,058 from 22 sites	***P-***value
Age (Median, IQR)		40.0 (33.0, 51.0)	40.0 (33.0, 50.0)	45.0 (35.0, 55.0)	< .001
Gender, n (%)	Male	2035 (22.8)	1793 (22.7)	242 (22.9)	0.926
	Female/Other	6906 (77.2)	6090 (77.3)	816 (77.1)	
Race, n (%)	Black/African American	383 (4.3)	348 (4.4)	35 (3.3)	0.003
	Other	795 (8.9)	726 (9.2)	69 (6.5)	
	White	7763 (86.8)	6809 (86.4)	954 (90.2)	
Hispanic or Latino Ethnicity, n (%)	Prefer not to answer	92 (1.0)	79 (1.0)	13 (1.2)	0.002
	Yes	550 (6.2)	510 (6.5)	40 (3.8)	
	No	8299 (92.8)	7294 (92.5)	1005 (95.0)	
Healthcare Environment, n (%)	Other	838 (9.4)	687 (8.7)	151 (14.3)	< .001
	Outpatient	826 (9.2)	679 (8.6)	147 (13.9)	
	Skilled Nursing Facility, Urgent Care, or Emergency Medical Services	107 (1.2)	87 (1.1)	20 (1.9)	
	Inpatient	7170 (80.2)	6430 (81.6)	740 (69.9)	
Role in Healthcare Setting, n (%)	Other	1418 (15.9)	1239 (15.7)	179 (16.9)	< .001
	Medical Assistant	411 (4.6)	359 (4.6)	52 (4.9)	
	Paramedic/Emergency Medical Technician	112 (1.3)	98 (1.2)	14 (1.3)	
	Dietary/Nutrition/Food Services, Environmental Services	155 (1.7)	125 (1.6)	30 (2.8)	
	Respiratory Therapist	116 (1.3)	106 (1.3)	10 (0.9)	
	Administrative staff	650 (7.3)	550 (7.0)	100 (9.5)	
	Physical therapist	139 (1.6)	102 (1.3)	37 (3.5)	
	Lab technician, Pharmacist, Pharmacy technician	454 (5.1)	390 (4.9)	64 (6.0)	
	Physician Assistant/Nurse Practitioner	557 (6.2)	504 (6.4)	53 (5.0)	
	Physician, Physician-in-training	1961 (21.9)	1769 (22.4)	192 (18.1)	
	Nurse (RN/LPN)	2890 (32.3)	2571 (32.6)	319 (30.2)	
	Missing	78 (0.9)	70 (0.9)	8 (0.8)	

Table 5 shows baseline characteristics of the study population stratified by number of beds of the affiliated hospital. Overall, participants from smaller hospitals tended to be older, were more likely to identify as White and less likely to identify as Hispanic and were less likely to be employed as physicians compared with participants from larger hospitals.

**Table 5:** Characteristics of participants and unadjusted outcome stratified by number of beds of employing healthcare facility

	Level (number of beds)	Overall *N* = 8941 from 97 sites	25–99 *N *= 143 from 4 sites	100–199 *N* = 387 from 11 sites	200–299 *N* = 387 from 13 sites	300–399 *N* = 652 from 9 sites	400–499 *N* = 404 from 6 sites	500 + *N* = 289 from 54 sites	***P-***value
Age (Median, IQR)		40.0 (33.0-51.0)	46.0 (38.0-56.0)	43.0 (35.0-52.0)	42.0 (35.0-53.0)	45.0 (36.0-55.0)	40.0 (32.0-50.0)	40.0 (33.0-50.0)	< .001
Gender, n (%)	Male	2035 (22.8)	25 (17.5)	69 (17.8)	159 (24.4)	89 (22.0)	63 (21.8)	1630 (23.1)	0.099
	Female	6906 (77.2)	118 (82.5)	318 (82.2)	493 (75.6)	315 (78.0)	226 (78.2)	5436 (76.9)	
Race, n (%)	Black/African American	383 (4.3)	2 (1.4)	11 (2.8)	26 (4.0)	10 (2.5)	37 (12.8)	297 (4.2)	< .001
	Other	795 (8.9)	8 (5.6)	22 (5.7)	51 (7.8)	27 (6.7)	34 (11.8)	653 (9.2)	
	White	7763 (86.8)	133 (93.0)	354 (91.5)	575 (88.2)	367 (90.8)	218 (75.4)	6116 (86.6)	
Hispanic or Latino Ethnicity, n (%)	Prefer not to answer	92 (1.0)	2 (1.4)	0 (0.0)	7 (1.1)	3 (0.7)	4 (1.4)	76 (1.1)	< .001
	Yes	550 (6.2)	2 (1.4)	17 (4.4)	23 (3.5)	19 (4.7)	30 (10.4)	459 (6.5)	
	No	8299 (92.8)	139 (97.2)	370 (95.6)	622 (95.4)	382 (94.6)	255 (88.2)	6531 (92.4)	
Healthcare Environment, n (%)	Other	838 (9.4)	14 (9.8)	38 (9.8)	69 (10.6)	24 (5.9)	12 (4.2)	681 (9.6)	< .001
	Outpatient	826 (9.2)	61 (42.7)	64 (16.5)	89 (13.7)	31 (7.7)	8 (2.8)	573 (8.1)	
	Skilled Nursing Facility, Urgent Care, or Emergency Medical Services	107 (1.2)	9 (6.3)	11 (2.8)	9 (1.4)	2 (0.5)	2 (0.7)	74 (1.0)	
	Inpatient	7170 (80.2)	59 (41.3)	274 (70.8)	485 (74.4)	347 (85.9)	267 (92.4)	5738 (81.2)	
Role in Healthcare Setting, n (%)	Other	1418 (15.9)	18 (12.6)	57 (14.7)	124 (19.0)	55 (13.6)	50 (17.3)	1114 (15.8)	< .001
	Medical Assistant	411 (4.6)	7 (4.9)	22 (5.7)	46 (7.1)	18 (4.5)	28 (9.7)	290 (4.1)	
	Paramedic/Emergency Medical Technician	112 (1.3)	4 (2.8)	1 (0.3)	6 (0.9)	6 (1.5)	4 (1.4)	91 (1.3)	
	Dietary/Nutrition/Food Services, Environmental Services	155 (1.7)	3 (2.1)	12 (3.1)	14 (2.1)	21 (5.2)	6 (2.1)	99 (1.4)	
	Respiratory Therapist	116 (1.3)	4 (2.8)	2 (0.5)	8 (1.2)	3 (0.7)	4 (1.4)	95 (1.3)	
	Administrative staff	650 (7.3)	8 (5.6)	25 (6.5)	42 (6.4)	41 (10.1)	47 (16.3)	487 (6.9)	
	Physical therapist	139 (1.6)	1 (0.7)	14 (3.6)	31 (4.8)	5 (1.2)	2 (0.7)	86 (1.2)	
	Lab technician, Pharmacist, Pharmacy technician	454 (5.1)	7 (4.9)	27 (7.0)	42 (6.4)	28 (6.9)	15 (5.2)	335 (4.7)	
	Physician Assistant/Nurse Practitioner	557 (6.2)	12 (8.4)	26 (6.7)	35 (5.4)	25 (6.2)	10 (3.5)	449 (6.4)	
	Physician, Physician-in-training	1961 (21.9)	33 (23.1)	53 (13.7)	96 (14.7)	60 (14.9)	26 (9.0)	1693 (24.0)	
	Nurse (RN/LPN)	2890 (32.3)	44 (30.8)	138 (35.7)	206 (31.6)	141 (34.9)	95 (32.9)	2266 (32.1)	
	Missing	78 (0.9)	2 (1.4)	10 (2.6)	2 (0.3)	1 (0.2)	2 (0.7)	61 (0.9)	

### Association between healthcare facility characteristics and access to personal protective equipment

There was no association either before or after adjustment (for age, gender, race, ethnicity, role in healthcare setting (e.g. nurse), healthcare environment (e.g. outpatient), and self-reported medical comorbidities) between any healthcare facility characteristic (ownership status, teaching status, number of beds, and metropolitan vs. micropolitan location) and access to personal protective equipment (Table 6).

**Table 6:** Unadjusted and adjusted odds ratios for the association between healthcare facility characteristics and outcomes for healthcare workers

	Unadjusted		Adjusted	
Parameter	Estimate (95% CI)	***P-***value	Estimate (95% CI)	***P-***value
**Access to Personal Protective Equipment Summary Score**				
** Bed Size (Reference = 25–99)**				
100–199	-0.01 (-0.54, 0.52)	0.962	0.16 (-0.64, 0.96)	0.692
200–299	0.16 (-0.38, 0.71)	0.558	0.42 (-0.29, 1.14)	0.246
300–399	-0.18 (-0.92, 0.56)	0.632	0.21 (-0.82, 1.25)	0.687
400–499	-0.42 (-1.37, 0.52)	0.379	-0.11 (-1.31, 1.09)	0.856
500 +	-0.34 (-0.88, 0.20)	0.221	-0.23 (-1.02, 0.57)	0.574
** Location (Micro vs. Metro)**	0.32 (-0.00, 0.64)	0.053	0.06 (-0.48, 0.60)	0.831
** Teaching Status (Yes vs. No)**	-0.15 (-0.74, 0.44)	0.616	-0.12 (-0.64, 0.40)	0.645
** Profit Type (Reference = Not-for-Profit)**				
For-Profit	0.27 (-0.36, 0.90)	0.403	0.08 (-0.65, 0.80)	0.832
Government	0.30 (-0.24, 0.85)	0.270	0.32 (-0.19, 0.83)	0.219
** COVID-19 Diagnosis**				
** Bed Size (Reference = 25–99)**				
100–199	1.42 (0.42, 4.88)	0.573	0.90 (0.32, 2.55)	0.849
200–299	2.10 (0.58, 7.61)	0.261	1.94 (0.68, 5.48)	0.214
300–399	1.90 (0.48, 7.54)	0.360	1.27 (0.37, 4.31)	0.703
400–499	2.17 (0.65, 7.22)	0.206	1.27 (0.45, 3.60)	0.648
500 +	1.18 (0.35, 3.94)	0.785	0.82 (0.30, 2.25)	0.707
** Location (Micro vs. Metro)**	0.79 (0.46, 1.38)	0.415	**0.42 (0.24, 0.71)**	**0.002**
** Teaching Status (Yes vs. No)**	0.78 (0.46, 1.33)	0.366	0.95 (0.66, 1.39)	0.808
** Profit Type (Reference = Not-for-Profit)**				
For-Profit	**1.94 (1.26, 3.01)**	**0.003**	**1.76 (1.02, 3.03)**	**0.042**
Government	0.83 (0.48, 1.42)	0.490	0.93 (0.55, 1.59)	0.804
** Burnout**				
** Bed Size (Reference = 25–99)**				
100–199	1.23 (0.61, 2.47)	0.5691	1.13 (0.48, 2.66)	0.781
200–299	0.76 (0.45, 1.27)	0.2887	0.77 (0.42, 1.41)	0.395
300–399	0.77 (0.41, 1.44)	0.4125	0.71 (0.33, 1.55)	0.390
400–499	1.27 (0.70, 2.32)	0.4319	1.23 (0.54, 2.81)	0.622
500 +	0.79 (0.50, 1.27)	0.3371	0.68 (0.34, 1.40)	0.297
** Location (Micro vs. Metro)**	0.92 (0.60, 1.42)	0.7146	0.80 (0.53, 1.22)	0.307
** Teaching Status (Yes vs. No)**	0.94 (0.74, 1.20)	0.6270	1.05 (0.84, 1.31)	0.655
** Profit Type (Reference = Not-for-Profit)**				
For-Profit	0.75 (0.49, 1.14)	0.1797	**0.53 (0.29, 0.98)**	**0.044**
Government	1.14 (0.89, 1.46)	0.3168	1.13 (0.87, 1.46)	0.369
** Depressive Symptoms**				
** Bed Size (Reference = 25–99)**				
100–199	0.83 (0.41, 1.69)	0.610	0.51 (0.25, 1.07)	0.075
200–299	0.79 (0.40, 1.59)	0.516	0.61 (0.32, 1.15)	0.128
300–399	1.04 (0.51, 2.14)	0.913	0.71 (0.34, 1.48)	0.362
400–499	0.77 (0.27, 2.14)	0.611	0.45 (0.15, 1.31)	0.142
500 +	0.87 (0.46, 1.66)	0.669	0.53 (0.27, 1.04)	0.067
** Location (Micro vs. Metro)**	**0.80 (0.66, 0.98)**	**0.028**	0.67 (0.43, 1.04)	0.075
** Teaching Status (Yes vs. No)**	1.05 (0.81, 1.36)	0.706	1.15 (0.89, 1.48)	0.288
** Profit Type (Reference = Not-for-Profit)**				
For-Profit	1.67 (0.96, 2.90)	0.067	1.82 (0.89, 3.71)	0.099
Government	1.15 (0.89, 1.48)	0.291	1.14 (0.87, 1.50)	0.325

Bolded rows indicate a statistically significant association

### Association between healthcare facility characteristics and COVID-19 diagnosis

Before adjustment, only for-profit ownership of the employing healthcare facility was associated with COVID-19 diagnosis (OR 1.94, 95% CI 1.26–3.01). After adjustment, this association was attenuated but was still significant (aOR 1.76, 95% CI 1.02–3.03). Furthermore, while before adjustment there was no association between micropolitan location of the employing healthcare facility and COVID-19 diagnosis (OR 0.79, 95% CI 0.46–1.38), after adjustment, healthcare workers employed by facilities located in micropolitan areas had lower adjusted odds of COVID-19 diagnosis (aOR 0.42, 95% CI 0.24–0.71). There was no association between number of beds or teaching status and COVID-19 diagnosis either before or after adjustment (Table 6).

## Associations between healthcare facility characteristics and burnout and depressive symptoms

Before adjustment, no healthcare facility characteristic was associated with burnout. However, after adjustment, for-profit ownership of the employing healthcare facility was associated with decreased odds of burnout (aOR 0.53, 95% CI 0.29–0.98) (Table 6). Before adjustment, participants employed by healthcare facilities in micropolitan areas had decreased odds of experiencing depressive symptoms (OR 0.80, 95% CI 0.66–0.98); however, after adjustment this association was no longer observed (aOR 0.67, 95% CI 0.43–1.04).

## Discussion

In this large, nationwide, longitudinal patient-reported outcomes study of nearly 9,000 healthcare workers, we found that working in a for-profit healthcare facility was associated with 76% greater odds of COVID-19 infection but 43% decreased odds of reporting burnout after controlling for demographics, role in the workplace, type of healthcare facility, and comorbid condition burden. We also found that working at a healthcare facility in a micropolitan area vs metropolitan area was associated with 68% decreased odds of COVID-19 infection.

Our study provides important information for public health systems. During infectious disease epidemics, health system resilience (defined as the ability of health systems to resist and adapt to external threats) is of paramount importance, and one critical dimension of health system resilience is having sufficient staffing to effectively run critical health services [17, 18]. Healthcare facilities vary substantially in their operational models, particularly in the United States, where the complex patchwork of reimbursement structures and concordant incentives for healthcare facilities results in sometimes dramatic differences in operational approaches [19–21]. Understanding how these different operational models intersect with experiences of healthcare workers is of paramount importance to developing effective public health approaches to pandemic preparedness [22–24].

Our study also has important implications for health system performance beyond infectious disease outbreaks. The literature exploring outcomes, especially health and psychosocial outcomes, of healthcare workers and how these outcomes vary according to the operational models of the facility by which they are employed is unfortunately sparse [25–28]. Future research is critically needed to understand how healthcare workers’ experiences may be affected by operational strategies used by their employing healthcare facilities. Given the incredibly high rates of burnout among patient facing staff in United States healthcare facilities and workforce shortages that are expected to continue to worsen in coming years, identifying whether particular operational models or workplace structures are particularly associated with adverse psychosocial outcomes for healthcare workers may allow policymakers and hospital administrators to identify the most effective targets for areas of intervention to improve retention and limit burnout among clinical staff [29, 30].

There are a broad range of possible explanations for the associations observed in this study. With regard to for-profit versus non-profit status, it is possible that for-profit healthcare facilities were less likely to support healthcare worker adherence to COVID-19 prevention strategies, perhaps related to decreased staffing levels, which prior studies have shown are more common in for-profit medical facilities [31, 32]. Furthermore, prior studies have shown that for-profit facilities in the United States experienced greater levels of financial instability than non-profit facilities, which could have resulted in increased strain on facility and staff resources leading to greater COVID-19 infection rates [33, 34].

The decreased rate of burnout seen in for-profit facilities is not consistent with prior studies before the COVID-19 pandemic. For example, a study in Sweden showed that burnout levels were the highest at a private, for-profit hospitals compared to a publicly administered hospital [35]. Furthermore, prior studies in the United States have shown that for-profit nursing homes tended to have worse results with regard to employee wellbeing [36]. However, these studies were conducted before the COVID-19 pandemic, so it is unclear the impact the pandemic may have had on burnout outcomes. It is also possible that our results are a result of unmeasured selection bias, given that the HERO study advertised participation most prominently in not-for-profit academic medical centers, meaning that participants from for-profit facilities may have been those with the most emotional reserve to participate in survey efforts beyond their work and therefore may have been less likely to report burnout.

Regarding the lower rate of COVID-19 diagnoses observed in healthcare workers employed by facilities located in micropolitan vs. metropolitan environments, it is possible that the underlying spread of COVID-19 during the study period may have been most prominent in metropolitan areas. Prior studies showed that in the first 5 months of the COVID-19 pandemic (during which the HERO study enrolled most participants), the incidence rates of COVID-19 cases were higher in metropolitan areas; incidence rates in non-metropolitan areas overtook incidence rates in metropolitan areas in approximately August 2020 [37]. Another study demonstrates that a much smaller number of micropolitan counties were classified as COVID-19 hotspots than metropolitan counties, providing further support to this interpretation [38].

Our study has limitations. One key limitation of our study is its reliance on self-reported data with risk for selection bias. Furthermore, our study only included sites where at least 10 healthcare workers responded to the survey, which systematically excluded the smallest sites, which plausibly could be systematically different than larger sites and could introduce a risk of bias. Furthermore, the HERO registry was coordinated through several large academic medical centers and therefore likely over-represents participants from academic medical centers. Our study also relies on American Hospital Association data to perform linkage, which, while effective at identifying hospitals and health systems, may not be as effective at identifying clinics, especially those that are independent of larger health systems. However, while these limitations may limit the generalizability of our study, our results are nonetheless meaningful for policymakers studying the healthcare facilities where most healthcare workers are employed. Another limitation of our study is our inability to control for local rates of COVID-19 cases at each presenting hospital, which could conceivably be a surrogate for the stress on local health systems. County-level data is insufficient for such a purpose, as the case rates at each presenting healthcare facility may not be closely related to the number of cases in the county the facility is located in. Estimating and evaluating variation in COVID-19 caseload at the level of individual healthcare facilities could be an important direction for future research. Furthermore, there are a variety of causal pathways that could connect our endpoints with each other (e.g. lack of access to PPE leading to COVID-19 infection leading to burnout leading to depression, or burnout leading to COVID-19 infection [through decreased use of appropriate PPE, if available]). Future studies should explicitly evaluate these questions using appropriate causal inference strategies.

Strengths of our study include its particularly large size (the HERO registry is the largest of its kind in the United States), reliance on participant reported outcomes that are directly relevant to healthcare workers, and our ability to adjust for key characteristics of healthcare workers such as their role in the healthcare workplace, age, and self-reported medical comorbidities.

## Conclusions

In summary, our work provides important preliminary data assessing the impact of healthcare facility structure and operational characteristics on healthcare worker outcomes during infectious disease pandemics. Future studies to carefully track the outcomes of healthcare workers and the association between these outcomes and operational characteristics of the facilities that employ them are urgently needed to inform health policy. Future analyses of the mechanisms of the association between healthcare facility characteristics and outcomes are needed to inform strategies to promote resilience against future pandemics and to promote stability and limit burnout in the healthcare workforce. Policymakers should be aware of the potential association between health system structure and healthcare facility characteristics and healthcare worker outcomes during an infectious disease pandemic.

### Supplementary Information

Supplementary Material 1.

## Data Availability

The data that support the findings of this study are available from the HERO registry, but restrictions apply to the availability of these data and so are not publicly available. Data are however available from the authors upon reasonable request and with permission of the HERO Registry oversight committee. Please contact Hero-Research@duke.edu to request access to the data.

## Appendix 1

### Dependent and independent variables used in the present study

**Table:**

Dependent Variables		
Variable Name	Survey Details	Variable Details
Access to personal protective equipment (PPE)	HERO registry personal protective equipment survey, which asked participants to rate “During the last day you were at work, how much of a problem did you have getting access to the following types of PPE: respiratory mask (e.g. N95, KN95, or other), surgical mask, powered air purifying respirator (PAPR), face shield/goggles, gloves, gowns, hand sanitizer, soap, and cleaning/disinfecting products.” Participants were asked to respond with “No problem,” “small problem,” “Big problem,” and “Not applicable to me.” Participants were also asked, “During the last day you were at work, for how many patients did you have to re-use the same N95/KN95 mask respirator or surgical mask when you would have otherwise used a new mask?” Responses to this question included “Did not have patient contact, none (1 mask per patient), some patients, most patients, and all patients).	Included as a summary score with values ranging from 0-21, with 0 representing the least difficulty with PPE access and 21 representing the highest difficulty with PPE acces
COVID-19 diagnosis	Self-report	Self-report of diagnosis with COVID-19
Burnout	Response to the burnout questionnaire from Rohland et al., 2004, which states, “Overall, based on your definition of burnout, how would you rate your level of burnout.” Participants are asked to respond with the following categories, “1) I enjoy my work. I have no symptoms of burnout; 2) Occasionally, I am under stress, and I don’t always have as much energy as I once did, but I don’t feel burned out; 3) I am definitely burning out and have one or more symptoms of burnout, such as physical and emotional exhaustion; 4) The symptoms of burnout that I’m experiencing won’t go away. I think about frustration at work a lot; 5) I feel completely burned out and often wonder if I can go on. I am at the point where I may need some changes or may need to seek some sort of help.”	Responses of 1 or 2 were classified as no burnout, and responses of 3, 4, or 5 were classified as burnout.
Depression	Responses to the PROMIS-T questionnaire (Pilkonis et al, 2011; Hays et al., 2009).	Scores >60 were classified as experiencing depression
**Independent Variables**		
Age	Self-reported	Continuous variable
Gender	Self-reported	Categorical variable, levels were Male and Female
Race	Self-reported	Categorical variable, levels were Black/African American, White, and Other
Ethnicity	Self-reported	Categorical variable, levels were Hispanic, non-Hispanic, and prefer not to answer
Role in healthcare setting	Self-reported	Categorical variable, levels were medical assistant, paramedic/emergency medical technician, dietary/nutrition/food services, environmental services, respiratory therapist, administrative staff, physical therapist, lab technician, pharmacist, pharmacy technician, physician assistant/nurse practitioner, physician, physician-in-training, nurse (RN/LPN), and other.
Category of healthcare environment	Self-reported	Categorical: Outpatient/Physical Therapy/Speech Therapy/Ambulatory Clinic/Rural Health clinic, Skilled nursing facility/nursing facility/urgent care/emergency services, hospital/inpatient.
Health conditions at baseline	Self-report	Binary categorical variables, one for each condition as follows: hypertension, diabetes, chronic obstructive pulmonary disease, asthma, smoking, kidney disease, and autoimmune disease.

## Appendix 2

### Study tables including frequencies of missing data, stratified by exposures of interest

**Table 7:** Missingness by facility bed size

	*Level*	*Overall* *N= 8941 from 97 sites*	*25-99* *N= 143 from 4 sites*	*100-199* *N= 387 from 11 sites*	*200-299* *N= 387 from 13 sites*	*300-399* *N= 652 from 9 sites*	*400-499* *N= 404 from 6 sites*	*500+* *N= 289 from 54 sites*	*P-value*
Age (Median, IQR)		40.0 (33.0 - 51.0)	46.0 (38.0 - 56.0)	43.0 (35.0 - 52.0)	42.0 (35.0 - 53.0)	45.0 (36.0 - 55.0)	40.0 (32.0 - 50.0)	40.0 (33.0 - 50.0)	<.001
	Missing (%)	(0.0)	(0.0)	(0.0)	(0.0)	(0.2)	(0.0)	(0.0)	
Gender, n (%)	Male	2035 (22.8)	25 (17.5)	69 (17.8)	159 (24.4)	89 (22.0)	63 (21.8)	1630 (23.1)	0.099
	Female/Other	6906 (77.2)	118 (82.5)	318 (82.2)	493 (75.6)	315 (78.0)	226 (78.2)	5436 (76.9)	
Race, n (%)	Other	795 (8.9)	8 (5.6)	22 (5.7)	51 (7.8)	27 (6.7)	34 (11.8)	653 (9.2)	<.001
	Black/African American	383 (4.3)	2 (1.4)	11 (2.8)	26 (4.0)	10 (2.5)	37 (12.8)	297 (4.2)	
	White	7763 (86.8)	133 (93.0)	354 (91.5)	575 (88.2)	367 (90.8)	218 (75.4)	6116 (86.6)	
Hispanic or Latino Ethnicity, n (%)	Prefer not to answer	92 (1.0)	2 (1.4)	0 (0.0)	7 (1.1)	3 (0.7)	4 (1.4)	76 (1.1)	<.001
	Yes	550 (6.2)	2 (1.4)	17 (4.4)	23 (3.5)	19 (4.7)	30 (10.4)	459 (6.5)	
	No	8299 (92.8)	139 (97.2)	370 (95.6)	622 (95.4)	382 (94.6)	255 (88.2)	6531 (92.4)	
Healthcare Environment, n (%)	Other	838 (9.4)	14 (9.8)	38 (9.8)	69 (10.6)	24 (5.9)	12 (4.2)	681 (9.6)	<.001
	Outpatient Physical Therapy/Speech Pathology/Ambulatory clinic/Rural Health Clinic	826 (9.2)	61 (42.7)	64 (16.5)	89 (13.7)	31 (7.7)	8 (2.8)	573 (8.1)	
	Skilled Nursing Facility/Nursing Facility/Urgent care clinic/Emergency services(medical,police,fire)	107 (1.2)	9 (6.3)	11 (2.8)	9 (1.4)	2 (0.5)	2 (0.7)	74 (1.0)	
	Hospital	7170 (80.2)	59 (41.3)	274 (70.8)	485 (74.4)	347 (85.9)	267 (92.4)	5738 (81.2)	
Role in Healthcare Setting, n (%)	Other	1418 (15.9)	18 (12.6)	57 (14.7)	124 (19.0)	55 (13.6)	50 (17.3)	1114 (15.8)	<.001
	Medical Assistant	411 (4.6)	7 (4.9)	22 (5.7)	46 (7.1)	18 (4.5)	28 (9.7)	290 (4.1)	
	Paramedic/Emergency Medical Technician	112 (1.3)	4 (2.8)	1 (0.3)	6 (0.9)	6 (1.5)	4 (1.4)	91 (1.3)	
	Dietary/Nutrition/Food Services, Environmental Services	155 (1.7)	3 (2.1)	12 (3.1)	14 (2.1)	21 (5.2)	6 (2.1)	99 (1.4)	
	Respiratory Therapist	116 (1.3)	4 (2.8)	2 (0.5)	8 (1.2)	3 (0.7)	4 (1.4)	95 (1.3)	
	Administrative staff	650 (7.3)	8 (5.6)	25 (6.5)	42 (6.4)	41 (10.1)	47 (16.3)	487 (6.9)	
	Physical therapist	139 (1.6)	1 (0.7)	14 (3.6)	31 (4.8)	5 (1.2)	2 (0.7)	86 (1.2)	
	Lab technician, Pharmacist, Pharmacy technician	454 (5.1)	7 (4.9)	27 (7.0)	42 (6.4)	28 (6.9)	15 (5.2)	335 (4.7)	
	Physician Assistant/Nurse Practitioner	557 (6.2)	12 (8.4)	26 (6.7)	35 (5.4)	25 (6.2)	10 (3.5)	449 (6.4)	
	Physician, Physician-in-training	1961 (21.9)	33 (23.1)	53 (13.7)	96 (14.7)	60 (14.9)	26 (9.0)	1693 (24.0)	
	Nurse (RN/LPN)	2890 (32.3)	44 (30.8)	138 (35.7)	206 (31.6)	141 (34.9)	95 (32.9)	2266 (32.1)	
	Missing	78 (0.9)	2 (1.4)	10 (2.6)	2 (0.3)	1 (0.2)	2 (0.7)	61 (0.9)	
Hypertension, n (%)	YES	965 (10.8)	21 (14.7)	45 (11.6)	65 (10.0)	51 (12.6)	59 (20.4)	724 (10.2)	<.001
	NO	7010 (78.4)	90 (62.9)	303 (78.3)	518 (79.4)	301 (74.5)	216 (74.7)	5582 (79.0)	
	Missing	966 (10.8)	32 (22.4)	39 (10.1)	69 (10.6)	52 (12.9)	14 (4.8)	760 (10.8)	
Diabetes, n (%)	YES	172 (1.9)	7 (4.9)	13 (3.4)	15 (2.3)	16 (4.0)	13 (4.5)	108 (1.5)	<.001
	NO	7803 (87.3)	104 (72.7)	335 (86.6)	568 (87.1)	336 (83.2)	262 (90.7)	6198 (87.7)	
	Missing	966 (10.8)	32 (22.4)	39 (10.1)	69 (10.6)	52 (12.9)	14 (4.8)	760 (10.8)	
Chronic obstructive pulmonary disease, n (%)	YES	21 (0.2)	1 (0.7)	3 (0.8)	3 (0.5)	1 (0.2)	1 (0.3)	12 (0.2)	0.099
	NO	7954 (89.0)	110 (76.9)	345 (89.1)	580 (89.0)	351 (86.9)	274 (94.8)	6294 (89.1)	
	Missing	966 (10.8)	32 (22.4)	39 (10.1)	69 (10.6)	52 (12.9)	14 (4.8)	760 (10.8)	
Asthma, n (%)	YES	946 (10.6)	17 (11.9)	40 (10.3)	60 (9.2)	55 (13.6)	41 (14.2)	733 (10.4)	0.069
	NO	7029 (78.6)	94 (65.7)	308 (79.6)	523 (80.2)	297 (73.5)	234 (81.0)	5573 (78.9)	
	Missing	966 (10.8)	32 (22.4)	39 (10.1)	69 (10.6)	52 (12.9)	14 (4.8)	760 (10.8)	
Smoking, n (%)	YES	235 (2.6)	4 (2.8)	18 (4.7)	31 (4.8)	10 (2.5)	11 (3.8)	161 (2.3)	<.001
	NO	7740 (86.6)	107 (74.8)	330 (85.3)	552 (84.7)	342 (84.7)	264 (91.3)	6145 (87.0)	
	Missing	966 (10.8)	32 (22.4)	39 (10.1)	69 (10.6)	52 (12.9)	14 (4.8)	760 (10.8)	
Kidney Disease, n (%)	YES	27 (0.3)	2 (1.4)	1 (0.3)	4 (0.6)	0 (0.0)	2 (0.7)	18 (0.3)	0.033
	NO	7948 (88.9)	109 (76.2)	347 (89.7)	579 (88.8)	352 (87.1)	273 (94.5)	6288 (89.0)	
	Missing	966 (10.8)	32 (22.4)	39 (10.1)	69 (10.6)	52 (12.9)	14 (4.8)	760 (10.8)	
Autoimmune Disease, n (%)	YES	352 (3.9)	9 (6.3)	17 (4.4)	25 (3.8)	16 (4.0)	12 (4.2)	273 (3.9)	0.561
	NO	7623 (85.3)	102 (71.3)	331 (85.5)	558 (85.6)	336 (83.2)	263 (91.0)	6033 (85.4)	
	Missing	966 (10.8)	32 (22.4)	39 (10.1)	69 (10.6)	52 (12.9)	14 (4.8)	760 (10.8)	

**Table 8:** Missingness by micropolitan vs metropolitan status

	*Level*	*Overall* *N= 8941 from 97 sites*	*Metro* *N= 8561 from 93 sites*	*Micro* *N= 380 from 4 sites*	*P-value*
Age (Median, IQR)		40.0 (33.0, 51.0)	40.0 (33.0, 51.0)	43.0 (35.0, 54.0)	0.002
	Missing (%)	(0.0)	(0.0)	(0.0)	
Gender, n (%)	Male	2035 (22.8)	1955 (22.8)	80 (21.1)	0.417
	Female/Other	6906 (77.2)	6606 (77.2)	300 (78.9)	
Race, n (%)	Other	795 (8.9)	775 (9.1)	20 (5.3)	<.001
	Black/African American	383 (4.3)	382 (4.5)	1 (0.3)	
	White	7763 (86.8)	7404 (86.5)	359 (94.5)	
Hispanic or Latino Ethnicity, n (%)	Prefer not to answer	92 (1.0)	87 (1.0)	5 (1.3)	<.001
	Yes	550 (6.2)	546 (6.4)	4 (1.1)	
	No	8299 (92.8)	7928 (92.6)	371 (97.6)	
Healthcare Environment, n (%)	Other	838 (9.4)	773 (9.0)	65 (17.1)	<.001
	Outpatient Physical Therapy/Speech Pathology/Ambulatory clinic/Rural Health Clinic	826 (9.2)	722 (8.4)	104 (27.4)	
	Skilled Nursing Facility/Nursing Facility/Urgent care clinic/Emergency services(medical,police,fire)	107 (1.2)	96 (1.1)	11 (2.9)	
	Hospital	7170 (80.2)	6970 (81.4)	200 (52.6)	
Role in Healthcare Setting, n (%)	Other	1418 (15.9)	1348 (15.7)	70 (18.4)	<.001
	Medical Assistant	411 (4.6)	375 (4.4)	36 (9.5)	
	Paramedic/Emergency Medical Technician	112 (1.3)	107 (1.2)	5 (1.3)	
	Dietary/Nutrition/Food Services, Environmental Services	155 (1.7)	145 (1.7)	10 (2.6)	
	Respiratory Therapist	116 (1.3)	111 (1.3)	5 (1.3)	
	Administrative staff	650 (7.3)	614 (7.2)	36 (9.5)	
	Physical therapist	139 (1.6)	133 (1.6)	6 (1.6)	
	Lab technician, Pharmacist, Pharmacy technician	454 (5.1)	421 (4.9)	33 (8.7)	
	Physician Assistant/Nurse Practitioner	557 (6.2)	535 (6.2)	22 (5.8)	
	Physician, Physician-in-training	1961 (21.9)	1896 (22.1)	65 (17.1)	
	Nurse (RN/LPN)	2890 (32.3)	2801 (32.7)	89 (23.4)	
	Missing	78 (0.9)	75 (0.9)	3 (0.8)	
Hypertension, n (%)	YES	965 (10.8)	929 (10.9)	36 (9.5)	0.449
	NO	7010 (78.4)	6712 (78.4)	298 (78.4)	
	Missing	966 (10.8)	920 (10.7)	46 (12.1)	
Diabetes, n (%)	YES	172 (1.9)	162 (1.9)	10 (2.6)	0.282
	NO	7803 (87.3)	7479 (87.4)	324 (85.3)	
	Missing	966 (10.8)	920 (10.7)	46 (12.1)	
Chronic obstructive pulmonary disease, n (%)	YES	21 (0.2)	18 (0.2)	3 (0.8)	0.021
	NO	7954 (89.0)	7623 (89.0)	331 (87.1)	
	Missing	966 (10.8)	920 (10.7)	46 (12.1)	
Asthma, n (%)	YES	946 (10.6)	901 (10.5)	45 (11.8)	0.352
	NO	7029 (78.6)	6740 (78.7)	289 (76.1)	
	Missing	966 (10.8)	920 (10.7)	46 (12.1)	
Smoking, n (%)	YES	235 (2.6)	218 (2.5)	17 (4.5)	0.018
	NO	7740 (86.6)	7423 (86.7)	317 (83.4)	
	Missing	966 (10.8)	920 (10.7)	46 (12.1)	
Kidney Disease, n (%)	YES	27 (0.3)	25 (0.3)	2 (0.5)	0.403
	NO	7948 (88.9)	7616 (89.0)	332 (87.4)	
	Missing	966 (10.8)	920 (10.7)	46 (12.1)	
Autoimmune Disease, n (%)	YES	352 (3.9)	336 (3.9)	16 (4.2)	0.732
	NO	7623 (85.3)	7305 (85.3)	318 (83.7)	
	Missing	966 (10.8)	920 (10.7)	46 (12.1)	

## Abbreviations

PPE: Personal protective equipment
HERO: Healthcare worker exposure, response, and outcomes
HCW: Healthcare worker
PCORI: Patient-centered outcomes research institute
